# Impaired non-verbal auditory memory maintenance in schizophrenia: An ERP study

**DOI:** 10.1016/j.scog.2025.100362

**Published:** 2025-04-17

**Authors:** Lei Liu, Wenyang Han, Juntao Yu, Lingna Lou, Dewen Zhou, Liang Li, Peng Xu, Feng Zou

**Affiliations:** a School of Psychological and Cognitive Sciences, Beijing Key Laboratory of Behavior and Mental Health, Peking University, Beijing 100871, China; b Department of Psychiatry, The 967th Hospital of PLA Joint Logistics Support Force, Dalian, China; c Faculty of Philosophy, Martin Luther University Halle-Wittenberg, Halle (Saale), Sachsen-Anhalt, Germany; d Institute of Psychology, Chinese Academy of Sciences, Beijing 100101, China

**Keywords:** Schizophrenia, Auditory working memory, Non-verbal auditory WM, ERP, Sustained anterior negativity

## Abstract

Individuals with schizophrenia (SZ) exhibit deficits in speech perception in noise, which are closely related to their abnormalities in auditory working memory (WM). Auditory WM, especially the non-verbal auditory WM, serves as a bridge between perception, action, and long-term memory, playing a crucial role in integrating sound sequences to facilitate auditory object perception and auditory scene analysis (ASA). Although considerable research has been conducted on auditory sensory memory and visual WM in schizophrenia, studies specifically addressing non-verbal auditory WM remain scarce. Therefore, this study recorded the behavioral performance and event related potentials of 36 SZ and 36 healthy controls (HC) during a modified non-musical tone-sequence delayed matching-to-sample task (DMTS). The results showed that, in the tone-sequence DMTS, SZ had not only lower accuracy but also slower reaction times compared to the HC. More importantly, during the retention period, the memory maintenance of SZ begins to decay rapidly from the mid-stage, manifested by a significantly reduction in the late sustained anterior negativity (SAN2). Meanwhile, the early sustained anterior negativity (SAN1) in patients showed a significant correlation with their general pathological symptoms. The pathological symptoms can be predicted by the SAN1 under load 4 condition. This study provides empirical evidence for the impairment of non-verbal auditory WM maintenance in schizophrenia, which is of significant importance for understanding the auditory dysfunction and ASA difficulties experienced by SZ.

## 1 Introduction

Schizophrenia is a chronic, severe, and highly disabling mental disorder characterized by significant cognitive impairments (Owen et al., 2016). Among these, deficits in working memory are one of the core features of cognitive dysfunction in schizophrenia (Forbes et al., 2009; Silver et al., 2003). Working memory (WM) is the ability to temporarily storage and manipulate information, acting as a bridge between perception, action, and long-term memory (Grimault et al., 2009), and playing a crucial role in our daily life. For instance, in a concert, only by remembering the notes you have just heard can you combine them with the subsequent notes to form a beautiful melody. Although previous research has accumulated substantial evidence on auditory sensory memory (Javitt et al., 1999; Javitt and Sweet, 2015) and visual WM in schizophrenia, there are few reports on auditory WM (Haigh et al., 2023). The auditory hierarchical processing model suggests that impairments in early auditory processing can impact higher-level cognitive processing (Dondé et al., 2023; Dondé et al., 2019; Zheng et al., 2021). There has been extensive research on auditory sensory memory deficits in schizophrenia; can we infer from this that there is also an abnormality in the patient's auditory working memory?

Unlike the parallel processing pattern of visual WM (which primarily deals with encoding and maintaining information about the shape, color, and location of objects), auditory WM requires the integration of auditory information across a continuous temporal dimension (a serial processing pattern) to facilitate auditory scene analysis, auditory object perception, and speech comprehension (Fernández-Rubio et al., 2022; Grimault et al., 2014). The temporal characteristics of auditory stimuli poses higher demands on the auditory WM system. In individuals with schizophrenia (SZ), impaired auditory WM can severely affect the maintenance of temporal sound signals, which in turn impacts their sound perception, speech recognition, social interaction. Investigating auditory WM impairments in SZ is crucial for understanding the mechanisms behind their auditory WM deficits. Such understanding is of significant importance for comprehending the auditory dysfunction, auditory scene analysis impairments (Middlebrooks et al., 2017) experienced by SZ.

Gold et al. (1997) first used the auditory Letter-Number Sequencing Test (LNST) to assess the auditory WM performance of patients with schizophrenia (Gold et al., 1997), and reported that patients not only exhibited impaired auditory WM but also showed a correlation between this impairment and their performance on the Wisconsin Card Sorting Task (WCST). Menon et al. (2001) used the 2-Back task to explore the functional and structural basis of auditory WM impairment in schizophrenia. They found that SZ performed significantly worse in the 2-Back task compared to healthy controls, and their reaction times were also significantly slower. More importantly, SZ showed a decline in WM-related activation in the left and right dorsolateral prefrontal cortices (DLPFC), frontal operculum, inferior parietal, and superior parietal cortices. The score of thinking disturbance symptom was negatively correlated with the activation of the right DLPFC. This suggests that the patients' thought disorder is related to the interruption of their WM processing (Menon et al., 2001). Seidman et al. (2012) found through the Auditory ACPT paradigm that not only SZ, but also individuals at high risk for schizophrenia showed impairment in auditory WM tasks (Seidman et al., 2012; Seidman et al., 2016). The aforementioned studies preliminarily revealed the abnormality of auditory WM in SZ. However, all the aforementioned studies used numbers or letters as materials, which cannot avoid the involvement of semantic and phonological encoding systems (Alunni-Menichini et al., 2014; Campo et al., 2024; Lefebvre et al., 2013), making it difficult to reflect the inherent processing mechanisms of auditory WM. To date, very few studies have specifically investigated the non-verbal auditory WM in SZ.

Explorations of non-verbal auditory memory typically employ the Sternberg paradigm (Sternberg, 1966) combined with non-musical tone stimuli (also known as the tone-sequence delayed matching-to-sample task, DMTS) (Lefebvre et al., 2013). Unlike familiar sounds such as speech or musical scales, non-musical tone stimuli do not belong to a standard scale and cannot be easily labeled or reformatted into other formal representations (Trehub et al., 1999). They are primarily stored in a low-level acoustic form (Lefebvre et al., 2013), which can minimize the activation of non-auditory short-term memory-related systems. In event-related potential (ERP) recordings based on the tone-sequence DMTS, memory maintenance induces a distinct sustained anterior negativity (SAN) in the frontal area (Lefebvre et al., 2013). Lefebvre et al. (2013) used a tone-sequence memory task and a control task with identical materials and parameters, except that in the memory task, participants had to judge whether the second sequence of tones matched the first tone sequence they heard; while in the control task, participants were instructed to completely ignore the first tone sequence and only judge the pitch changes of the last two tones in the second tone sequence. The results showed a pronounced SAN in the frontal area during the memory task; this SAN was not evident under the same auditory stimulation in the control condition (cf., Coffman et al., 2018; Coffman et al., 2016). Moreover, the SAN in the memory task increased with the load, and could effectively predict individual performance differences. Subsequent studies have confirmed that the SAN indeed effectively reflects the memory maintenance of low-level tone stimuli (Grimault et al., 2009; Grimault et al., 2014; Lefebvre and Jolicœur, 2016; Lefebvre et al., 2013; Nolden et al., 2013a; Nolden et al., 2013b; Simal and Jolicoeur, 2020).

To better capture the information maintenance and updating involved in WM, and simultaneously controlling the impact of encoding strategies based on tonal sequence contours, Lefebvre and Jolicœur (2016) randomly inserted white noise into the memory tone sequences and asked the participants to ignore the interference of the white noise, matching the probe tone sequence with the memory tone sequence extracted from the noise (Lefebvre and Jolicœur, 2016). Using this modified tone-sequence DMTS task to investigate the maintenance mechanisms of simple tone items in auditory WM, Lefebvre and Jolicœur (2016) found that, on the one hand, consistent with previous studies, the memory maintenance induced a significant frontal SAN component compared to the control condition (load 0). On the other hand, unlike previous studies, the maintenance effect of SAN (i.e., the increase of SAN with increasing load) was more pronounced in the early retention phase (330–1100 ms, SAN1) than in the later phase (1100–1900 ms, SAN2). Moreover, the SAN1 slope showed a slightly greater correlation with memory capacity compared to the SAN2 slope. This suggests that the load effect may reach a stable state in the later stage of the retention phase. To explore the behavioral and ERP related changes in the maintenance of non-verbal auditory WM in SZ, we attempt to use this modified tone-sequence DMST task combined with EEG recordings. Based on the aforementioned findings, we selected the retention interval of 330 to1110 ms as SAN1 and 1100 to 1900 ms as SAN2 for examination. We infer that, in the early stage of the memory retention, the SAN1 of SZ can effectively represent memory maintenance and individual difference, thus being more capable of discovering the association between SAN1 and behavioral performance as well as symptoms (cf., Lefebvre and Jolicœur, 2016). In the later stage of the retention phase, the patient's memory capacity declines rapidly, and SAN2 quickly returns to baseline level, which is not only significantly smaller than that of healthy controls but also difficult to associate with behavioral performance.

In summary, the current study aims to investigate the processing characteristics of non-verbal auditory WM maintenance between patients with schizophrenia and healthy controls using a modified tone-sequence DMTS task combined with EEG recordings. Drawing on existing research on auditory sensory memory and visual WM in schizophrenia, as well as models of auditory hierarchical processing, we hypothesize that SZ have deficits in the maintenance of non-verbal auditory WM. Specifically, patients with schizophrenia may exhibit longer reaction times and lower accuracy at the behavioral level. Meanwhile, SAN1 in the early stage of their memory retention may be associated with the clinical symptoms, while SAN2 in the late stage of memory retention may exhibit a significant decrease.

## 2 Materials and methods

### 2.1 Participants

We recruited thirty-six individuals diagnosed with schizophrenia according to DSM-V and thirty-six healthy controls (HC), all aged between 18 and 49 years, from the 967th Hospital and the local communities, respectively (**see Table1**). The diagnosis followed the Diagnostic and Statistical Manual of Mental Disorder-fifth Edition, DSM-V (American Psychiatric Association, 2013). Schizophrenia symptoms were assessed using the Positive and Negative Syndrome Scale (PANSS). All the patients received antipsychotic medication during the study period. They were right-handed, had a junior high school education or above, without abnormal intelligence. None of the healthy participants had a history of Axis-I psychiatric disorders defined by DSM-IV. Participants with hearing (show any pure-tone hearing impairments for each ear at the routine hearing screening frequencies of 500, 1000, 2000, 4000, and 6000 Hz) or vision loss, nervous system disease, alcohol or drug abuse, and treatment of the electroconvulsive therapy (ECT), TMS or tDCS within the past three months were excluded. Smoking was discontinued the day before the experiment, and written informed consent was signed before the experiment began. Subjects were compensated for their participation after the experiment finished. The study was approved by the Independent Ethics Committee of the 967th Hospital of the PLA Joint Logistics Support Force and the Committee for the Protection of Human and Animal Subjects at the School of Psychological and Cognitive Sciences, Peking University.

### 2.2 Materials and task

**Fig. 1:**
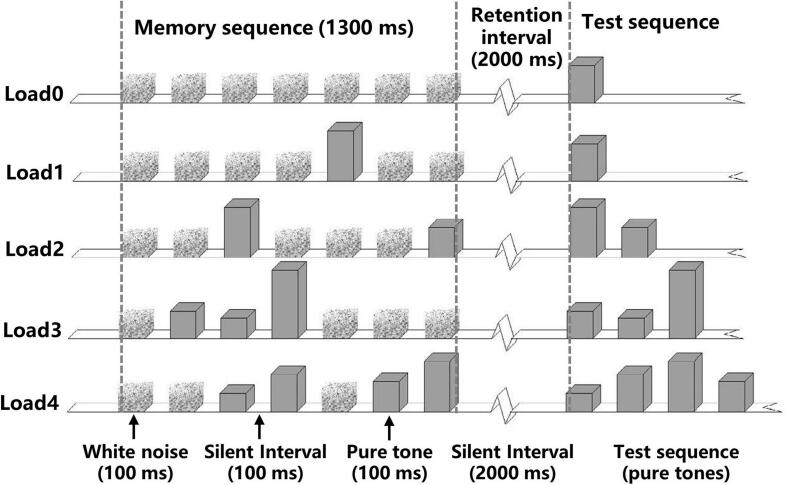
Example of the tone-sequence delayed matching-to-sample task (DMST). The DMST task encompasses three distinct stages: the encoding phase, which corresponds to the memory sequence and lasts for 1300 milliseconds; the maintenance phase, characterized by a 2000-millisecond silent retention interval during which the SAN is measured; and the retrieval phase, which corresponds to the test sequence. Pure tones are represented by the gray columns, with different heights indicating different frequencies; white noise sounds are represented by pixelated columns. In each condition (Load 0,1,2,3, and 4), trials begin with a 100 ms noise burst, followed by six additional sounds, which included a variety of noise bursts and pure tones, corresponding to different memory loads. After the maintenance phase, the second pure tone sequences are presented, participants have 3000 ms to judge whether the second tone sequence is the same as the previous one.

We employed a tone-sequence DMTS task combined with EEG recordings to investigate the changes of non-verbal auditory WM in SZ (**See** Fig. 1). The auditory stimuli were the same as those used in Lefebvre and Jolicœur (2016), consisting of white noise bursts and 14 pure tones spanning two octaves within the middle range of frequencies (i.e., 380, 419, 463, 511, 564, 623, 688, 759, 888, 925, 1022, 1128, 1245, 1375 Hz). All stimuli were generated using Matlab, with a sampling rate of 44,100 Hz, a duration of 100 ms, 10 ms rise and fall times. They were presented at 65 dB SPL via insert earphones (ER-3, Etymotic Research, Elk Grove Village, II). The entire experiment included 5 blocks, with each block containing 60 trials, and five auditory load conditions with 12 trials for each condition, presented randomly. After the experiment commenced, a fixation point was displayed on the screen for approximately 500 ms. Subsequently, a noise burst was presented binaurally to the subject, accompanied by six other sounds. Each sound was separated by a 100 ms silent interval, resulting in a total duration of 1300 ms for the first sequence of sound stimuli (referred to as Tone Sequence 1).

Under load 0 condition, all sounds are white noises, serving as the control condition. Under load 1 condition, one of the six white noises is replaced by a tone, which randomly appears in any of the six positions. Under load 2 condition, two white noises are replaced by tone stimuli. Load 3 and Load 4 conditions follow the same pattern accordingly. After an interval of 2 s, a tone sequence without white noise is presented (i.e., Tone sequence 2). In half of the trials, Tone sequence 2 is the same as Tone sequence 1; in the other half, Tone sequence 2 is altered and different from Tone sequence 1. Consequently, the duration of Tone sequence 2 ranges between 100 and 700 ms (**See** Fig. 1). The participant's task is to judge whether the second tone sequence is the same as the previous one by pressing the buttons. As a control condition, under Load 0, participants have no memory load because there is no association between Tone sequence 1 and Tone sequence 2. Participants are only need to listen to the tone stimulus in sequence 2 and make a “different” response. Participants have a maximum of 3 s to make a button response. Following the participant's response or after 3 s, there will be an 800 ms interval with a blank screen before proceeding to the next trial. After each block, participants can rest for 3 to 5 min. There are 60 trials for each condition, totaling 300 trials, with a duration of approximately 40 min.

### 2.3 EEG recordings

EEG signals were captured using a Neuroscan system (Curry 7, Compumedics Neuroscan, Inc., Charlotte, NC, USA) with a 64-electrode cap, adhering to the 10–10 international electrode placement system. The ground electrode was situated between FPz and Fz, and the online reference electrode was placed between Cz and CPz. The impedance between each electrode and the scalp was kept under 5 kΩ. The EEG recording was sampled at a rate of 500 Hz. The experiment was conducted in a sound-attenuated room with relatively moderate light.

### 2.4 Event-related potential analysis

EEG data were preprocessed using EEGLAB v14.1b (Delorme and Makeig, 2004). Initially, a FIR filter was applied for band-pass filtering between 0.1 and 30 Hz, and a 50 Hz notch filter was used to reduce potential line noise interference. Then, the data were manually inspected to remove segments with obvious artifacts such as head movements, muscle activity, sudden drifts, and jumps. Independent component analysis (ICA) was employed to correct for blinks, saccades, and other systematic artifacts (Delorme and Makeig, 2004). After artifact correction, the data were re-referenced to global average reference. The continuous data were segmented from 200 ms before to 4800 ms after stimulus onset, and baseline-corrected using the 200 ms pre-stimulus interval. The segmented data were further scrutinized using a sliding window peak-to-peak method (window width, 200 ms, window step, 100 ms, threshold 80 μV) (Lopez-Calderon and Luck, 2014; Luck, 2014) to exclude trials with amplitude changes exceeding ±80 μV, and then averaged according to experimental conditions. If the number of artifact trials for a single subject exceeded 35 % of the total number of trials, the data from that subject were excluded. Four patients with schizophrenia and six healthy subjects were excluded due to poor EEG recording or excessive ERP artifacts (invalid trials exceeding 35 %). A total of 32 valid patients with schizophrenia and 30 healthy volunteers were included in the data comparison. There were no significant differences in the number of valid trials across the four conditions. The memory maintenance related SAN1 (330–1110 ms after the onset of memory retention, or 1630 to 2400 ms after the stimulus onset) and SAN2 (1100–1900 ms after the onset of memory retention, or 2400 to 3200 ms after the stimulus onset) were extracted using the ERPLAB 10.0 toolbox (Lopez-Calderon and Luck, 2014).

### 2.5 Statistical analysis

Statistical analyses were conducted using JASP 0.19.0.0 (https://jasp-stats.org/) and IBM SPSS 20.0 (IBM Corp., Armonk, NY, USA). The normality was assessed by the Shapiro-Wilk test; the homogeneity of variances was examined using the Levene test. In repeated measures analysis of variance (ANOVA), when the assumption of sphericity is not met, the Greenhouse-Geisser method is used for correction. Post-hoc multiple comparisons were adjusted with Bonferroni correction. Cohen's d, partial eta-squared (*η*^*2*^*p*), and Fisher's z were utilized to describe the effect size of the *t*-test, ANOVA, and correlation analyses, respectively.

## 3 Results

### 3.1 Behavioral results

The basic information of the participants is presented in Table 1, with the two groups differing only in age. To eliminate the potential influence of age, we calculated the *Z*-scores of the age and included it as a covariate in both the repeated measures ANOVA and correlation analysis. We conducted a mixed-design (group x load) repeated measures analysis of covariance (ANCOVA) to explore the differences in auditory WM performance between SZ and HC in a tone-sequence DMTS task.

**Table 1:** Demographic and clinical characteristics for participants with schizophrenia (*N* = 36) and healthy controls (N = 36).

Characteristic	SCH (n = 36) Mean (SD)	HC (n = 36) Mean (SD)	*t*	*p*
Age (years)	27.611 (6.221)	23.611 (3.092)	−3.455	0.001
Female % (n)	NA	2.778 (1)	NA	NA
Education (years)	14.00 (2.23)	14.333 (1.568)	0.734	0.466
Ill duration	3.444 (4.878)	NA	NA	NA
Age of Onset (years)	24.167 (5.028)	NA	NA	NA
PANSS Total	58.083 (9.262)	NA	NA	NA
Positive	14.056 (5.149)	NA	NA	NA
Negative	15.083 (5.547)	NA	NA	NA
General	28.944 (4.928)	NA	NA	NA
Medication % (n)				
Olanzapine	50(18)	NA	NA	NA
Aripiprazole	38.89 (14)	NA	NA	NA
Risperidone	22.22 (8)	NA	NA	NA
Clozapine	13.89 (5)	NA	NA	NA

*Note*: Values are mean (SD) or % (n) as appropriate. SD, standard deviation; PANSS, positive and negative syndrome scale; Medication, reflects the number and percentage of individuals taking the corresponding medication. NA, not applicable.

**Fig. 2:**
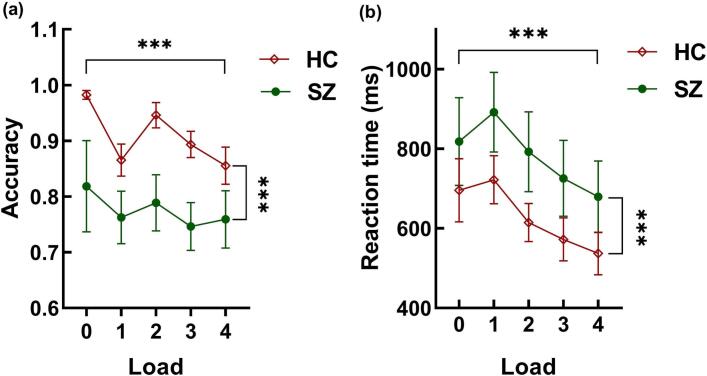
Behavioral performance of individuals with schizophrenia in non-verbal auditory working memory task. (a) The accuracy of the tone-sequence DMST task revealed significant group differences and load effects; (b) The reaction times of the tone-sequence DMST task revealed significant group differences and memory load effects, *p* < 0.001. ***, *p* < 0.001.

The ANCOVA for accuracy revealed a significant main effect of the group, *F*_(1,69)_ = 29.942, *p* < 0.001, *η*^*2*^_*p*_ = 0.303, with HC performing significantly better than SZ (Fig. 2). The load factor also showed a significant main effect, *F*_(2.003, 138.165)_ = 18.538, *p* < 0.001, *η*^*2*^_*p*_ = 0.212, with accuracy changing with load in a very interesting way: initially, the highest accuracy was observed under the control condition (load 0). Then, accuracy dropped significantly under the load 1 condition, reached the second-highest level under the Load 2 condition, and then declined with increasing load. Further post-hoc tests revealed that Load 0 significantly differed from the other four load conditions, *ts* ≥ 2.777, *ps* ≤ 0.023, *Cohen's d* ≥ 0.299. There was a clear difference between Load 1 and Load 2, *t*_(70)_ = −3.870, *p* < 0.001, *Cohen's d* = −0.416. Significant differences were also found between Load 2 and Load 3 (*t*_(70)_ = 3.302, *p* = 0.005, *Cohen's d* = 0.355), as well as between Load 2 and Load 4 (*t*_(70)_ = 4.323, *p* < 0.001, *Cohen's d* = 0.465). These results, on one hand, reflect the effect of the memory load, with a noticeable decline in subjects` accuracy from load 2 as the load increases; on the other hand, the lower accuracy under load 1 also demonstrates the significant disruptive effect of white noise stimulation. The interaction between load and group was not significant, *F*_(2.002, 138.165)_ = 2.227, *p* = 0.112, *η*^*2*^_*p*_ = 0.031.

The ANCOVA analysis for reaction time indicated a significant main effect of the group, *F*_(1, 69)_ = 4.250, *p* = 0.043, *η*^*2*^_*p*_ = 0.058, with reaction times of SZ being significantly longer than those of the HC (Fig. 2). The main effect of the load was also significant, *F*_(1.691，116.647)_ = 29.210, *p* < 0.001, *η*^*2*^_*p*_ = 0.297, suggesting that from load 1, participants' reaction times showed a decreasing trend with increasing load. There was no significant interaction between group and load, *F*_(1.691, 116.647)_ = 1.277, *p* = 0.279, *η*^*2*^_*p*_ = 0.018. Further post-hoc tests indicated that there were significant differences between Load 0 and Load 2, Load 3, Load 4, *ts* ≥ 2.681, *ps* ≤ 0.023, *Cohen's d* ≥ 0.259; between Load 1 and Load 2, Load 3, Load 4, *ts* ≥ 4.605, *ps* ≤ 0.001, *Cohen's d* ≥ 0.445; and between Load 2 and Load 3 (*t*_(70)_ = 2.780, *p* = 0.023, *Cohen's d* = 0.269), as well as Load 4 (*t*_(70)_ = 4.723, *p* < 0.001, *Cohen's d* = 0.457). Consistent with the results of accuracy, the longest reaction times under Load 1 seem to reveal the significant disruptive effect of the white noise, while the decreasing trend in reaction times following Load 1 with increasing load reveals the individual's contour-based encoding characteristics.

### 3.2 ERP results

**Fig. 3:**
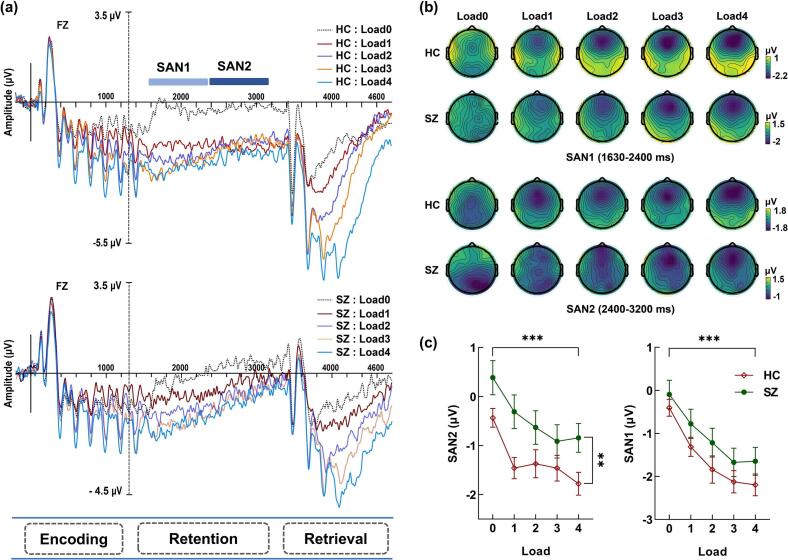
The grand averaged ERP waveforms for HC and SZ in the five load conditions. During the initial 1300 ms of stimulus encoding, seven distinct N1s were elicited in the frontal region by seven auditory stimuli. Subsequently, during the maintenance phase of WM, a pronounced sustained anterior negativity (SAN) was observed across all load conditions except for Load 0. Furthermore, during the retrieval and matching phase of WM, the probe stimuli under different load conditions elicited varying numbers of N1s, with Probe 1 to Probe 4 eliciting 1 to 4 N1s respectively. (b) Scalp voltage topographical maps revealed the distribution of SAN components induced by memory maintenance under five different load conditions. Both SAN1 (1630–2400 ms) and SAN2 (2400–3200 ms) demonstrated a distinct frontal distribution. (c) The mean amplitude of the frontal SAN2 demonstrated significant group difference and load effect; The mean amplitude of the frontal SAN1 only exhibited a significant load effect. ***, *p* < 0.001.

The current ERP waveforms have well replicated the findings of Lefebvre and Jolicœur (2016). From the waveforms and topographic maps of the healthy group under four load conditions (as shown in Fig. 3**a** and **b**), it can be seen that during the 1300 ms encoding phase of the stimulus, seven auditory stimuli elicited seven distinct N1s in the frontal area. Then, during the retention phase of WM, except for the Load 0 condition, all other load conditions elicited a very pronounced sustained anterior negativity (SAN). Furthermore, during the retrieval and matching phase of WM, probe stimuli under different load conditions elicited varying numbers of N1s, with probes 1 to 4 eliciting 1 to 4 N1s, respectively. Both the HC and the SZ showed very pronounced SAN components in the frontal area. SAN1 is from 330 to 1100 ms after the start of the retention period (i.e., 1630 to 2400 ms after the stimulus onset), and SAN2 is from 1100 to 1900 ms after the start of the retention period (i.e., 2400 to 3200 ms after the stimulus onset).

***Retention SAN1*** We selected the frontal SAN1 (the average of F1, Fz, F2, FC1, FCz, FC2) for a two-way ANCOVA (Fig. 3c). The results showed that the main effect of the load factor was significant, *F*_(3.263，211.805)_ = 33.613, *p* < 0.001, *η*^*2*^_*p*_ = 0.363, as the load level increased, SAN1 exhibited a significantly more negative trend. However, the group difference was not significant, *F*_(1,59)_ = 1.128, *p* = 0.292, *η*^*2*^_*p*_ = 0.019. There was no significant difference between the HC and SZ in the SAN1. The interaction between load and group was also not significant, *F*_(3.263，192.504)_ = 0.321, *p* = 0.826, *η*^*2*^_*p*_ = 0.005. Further post-hoc tests revealed that there were significant differences between Load 0 and Load 1 to Load 4, *ts* > 4.679, *ps* < 0.001, *Cohen's d* > 0.479, between Load 1 and Load 2 to Load 4, (*ts* > 2.820, *ps ≤* 0.021, *Cohen's d* > 0.289), and between Load 2 and Load 3, *t*_(61)_ = 2.926, *p* = 0.049, *Cohen's d* = 0.224. There was no significant difference between Load 3 and Load 4, *t*_(61)_ = 0.156, *p* = 1.000, *Cohen's d* = 0.014.

***Retention SAN2*** We further performed a two-way ANCOVA on the frontal SAN2. The results revealed a significant difference in the group factor, *F*_(1,59)_ = 4.597, *p* = 0.036, *η*^*2*^_*p*_ = 0.072; the HC exhibited a significantly more negative SAN2 compared to SZ. The main effect of the load was also significant, *F*_(3.206，189.172)_ = 16.738, *p* < 0.001, *η*^*2*^_*p*_ = 0.221, with SAN2 becoming more negative as the load level increased, a result that was also clearly visible in the EEG topographic maps. The interaction between load and group was not significant, *F*_(3.206，189.172)_ = 0.583, *p* = 0.638, *η*^*2*^_*p*_ = 0.010 (as shown in Fig. 3c). Further post-hoc tests indicated that there were significant differences between the control condition (Load 0) and the experimental conditions Load 1 to Load 4 (*ts* > 4.506, *ps* < 0.001, *Cohen's d* > 0.521). Additionally, there was a marginal significance between Load 1 and Load 4 (*t*_(61)_ = 2.778, *p* = 0.073, *Cohen's d* = 0.259), and the other conditions showed a trend of becoming more negative with increasing load, but there were no statistical differences (*ts* < 2.236, *ps* > 0.291, *Cohen's d* < 0.188). After removing the control condition Load 0, there was a significant difference between Load 1 and Load 4, (*t*_(61)_ = 2.778, *p* = 0.044, *Cohen's d* = 0.257), further confirming the existence of differences between the Load 1 and Load 4 conditions. The aforementioned results suggest that the amplitude of SAN shows a significantly more negative trend as the load increases, with the load effect primarily manifested in SAN1, while group differences are mainly reflected in SAN2.

**Fig. 4:**
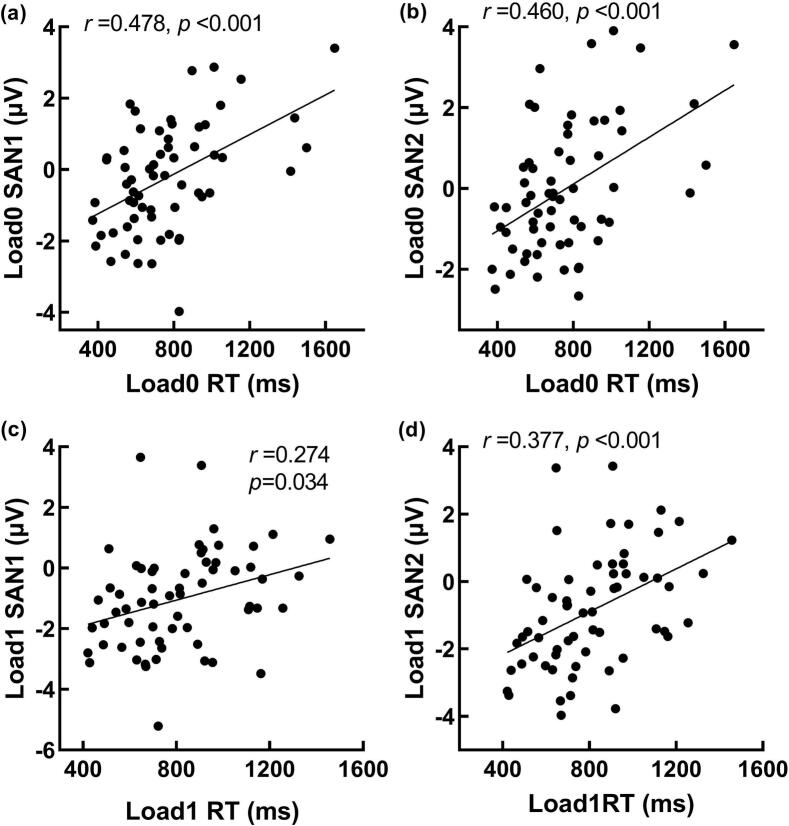
Scatter plots of reaction times with SAN1 and SAN2 under load0 and load1 conditions. (a) Significant partial correlation between reaction times and SAN1 under Load 0 conditions, *r* = 0.478, *p* < 0.001; (b) Significant partial correlation between reaction times and SAN2 under load 0 conditions, *r* = 0.460, *p* < 0.001; (c) Significant partial correlation between reaction times and SAN1 under Load 1 conditions, *r* = 0.274, *p* = 0.034; (d) Significant partial correlation between reaction times and SAN2 under load 1 conditions, *r* = 0.377, *p* < 0.001. ***, *p* < 0.001.

**Fig. 5:**
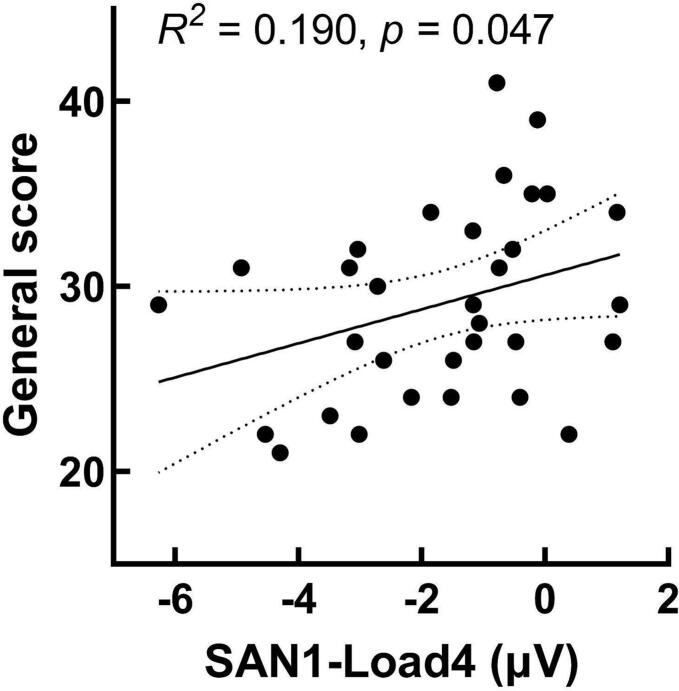
Significant partial correlations between SAN1-load 4 and general psychopathology symptom scores, *r* = 0.358, *p* = 0.048, *Fisher's z* = 0.375 (*rho* = 0.365, *p* = 0.043). SAN1-load4 has a significant predictive effect on the patients' pathological symptoms and cognitive dysfunction, *R*^*2*^ = 0.190, *p* = 0.047.

To explore the association between behavioral performance, ERP responses, and schizophrenia symptoms, we conducted partial correlation analyses between behavioral performance and SAN, as well as between SAN and the PANSS scores. The results showed that, after controlling for age and group, SAN1 and SAN2 during the retention phase were both significantly correlated with reaction times under Load 0 and Load 1 conditions, *rs* ≥ 0.274, *ps* ≤ 0.034, *Fisher's z* ≥ 0.282 (see Fig. 4). More importantly, in SZ, there was a significant partial correlation between SAN1 under load4 condition (Load4-SAN1) and general psychopathology symptom scores (GPS), *r* = 0.358, *p* = 0.048, *Fisher's z* = 0.375. Further regression analysis with Load4-SAN1 as the predictor variable, age as the covariate, and GPS as the dependent variable showed that Load4-SAN1 (*t* = 2.067, *p* = 0.048, 95%CI [0.01, 1.929]) significantly predicted the GPS (see Fig. 5), while the age factor (*t* = 1.710, *p* = 0.098, 95%CI [−0.046, 0.518]) did not effectively predict the GPS. The fitted model is (GPS = 24.129 + 0.97*Load4-SAN1 + 0.236*Age). The overall regression is significant (*R*^*2*^ = 0.190, adjusted *R*^*2*^ = 0.134, *F*_(2,31)_ = 3.394, *p* = 0.047).

## 4 Discussions

Auditory working memory (WM) refers to the capacity to briefly retain and manipulate auditory information. A limited number of studies have preliminarily explored auditory WM abnormalities in schizophrenia using auditory N-Back, ACPT tasks, and LNST tests. However, these studies, on one hand, cannot rule out the influence of language or speech encoding systems, and thus do not reflect the inherent characteristics of auditory WM. On the other hand, they only provide a simple assessment of the patients' WM performance without further in-depth revelation. Based on this, the current study employs a modified tone-sequence delayed matching task combined with EEG recordings to investigate the behavioral performance and electrophysiological correlates of non-verbal auditory WM deficits in SZ.

Consistent with the findings of Menon et al. (2001) and Seidman et al. (2012), our study revealed that SZ not only had lower accuracy but also slower reaction time in tone-sequence DMTS task. In Menon et al. (2001), participants were required to complete a 2-back continuous performance task, where numbers from 0 to 9 were presented through a female voice with an inter-stimulus-interval (ISI) of 2 s (Menon et al., 2001). Under WM condition, participants were asked to determine whether the currently presented number was the same as the one presented before two stimuli. In the control condition, participants were asked to respond immediately upon hearing the number “3”. The results showed that, compared with the control group, SZ not only had longer reaction times, but also had lower behavioral performance. Seidman et al. (2012) manipulated high and low loads as well as the presence or absence of interference factors in the ACPT paradigm. Participants were asked to respond to the stimulus “A” following a cue stimulus “Q” under various load and interference conditions (Seidman et al., 2012). The results showed that SZ performed worse than the HC in nearly all load and interference conditions. Together with the aforementioned studies, we further confirmed that SZ have impaired non-verbal auditory WM maintenance through a tone-sequence DMTS task with non-musical tone stimuli. Moreover, the memory maintenance deficit for non-musical tones may be further reflected in the abnormal processing of auditory digit N-back and letter ACPT tasks. The impaired maintenance of non-musical tone memory may indicate a fundamental WM deficit in maintaining pure auditory information, which is independent of both the semantic and phonetic encoding systems. Such a deficit is often a manifestation of insufficient WM capacity or unstable maintenance mechanisms, showing limited brain capacity for retaining unstructured sound information in the short term. For SZ, this deficit may be a direct manifestation of structural or functional issues in their auditory WM system. Besides, The abnormalities in early auditory processing, auditory sensory memory (Javitt, Liederman, Cienfuegos and Shelley, 1999, Javitt, Lee, Kantrowitz and Martinez, 2018; Javitt and Sweet, 2015; Liu et al., 2025), and auditory working memory in patients with schizophrenia not only support the auditory hierarchical processing model (Dondé, Silipo, Dias and Javitt, 2019, Dondé et al., 2023), but may also be the mechanism underlying their abnormal auditory scene analysis. All of these ultimately lead to their deficits in auditory cognitive function and difficulties in social interaction.

As we expected, SZ exhibit a clear SAN component during the memory maintenance phase, but in contrast to the healthy group, the SAN in patients gradually returns to baseline levels in the later stages of the maintenance phase, supporting the notion of rapid decay in their memory maintenance. Although no schizophrenia-related studies have yet revealed similar findings, a recent study on syllable N-back WM task in healthy aging individuals found that older adults showed significantly smaller frontal sustained negative potentials in the frontal region compared to younger controls, both in 1-Back and 2-Back tasks (Nowak et al., 2021). Studies on visual working memory in schizophrenia have also observed similar patterns. Leonard et al. (2013) investigated changes in working memory capacity in schizophrenia through a change detection task requiring the storage of multiple objects in working memory. They found that individuals with schizophrenia had significantly smaller amplitudes of the CDA component (also known as the posterior sustained negative wave, NSW, a sustained negative wave evoked during the retention phase of visual working memory that appears at the parietal region) under load 3 and load 5 conditions compared to healthy individuals (Leonard et al., 2013). These findings are consistent with our experimental results. It is generally believed that the late retention stage is more reflective of information maintenance (Grimault et al., 2014; Lefebvre and Jolicœur, 2016; Lefebvre et al., 2013), and the rapid decline of SAN2 in SZ suggests that the decrease in its non-verbal auditory memory is primarily caused by the decline in memory maintenance.

Further partial correlation analysis reveals that SAN1 and SAN2 during the retention phase both have significant partial correlations with reaction times under Load 0 and Load 1 conditions. These results align with the findings of Lefebvre and Jolicœur (2016). More importantly, in SZ, there is a significant partial correlation between SAN1-Load4 and general psychopathology scores. The smaller (more negative) the average amplitude of SAN1, the lower the general psychopathology scores. Moreover, SAN1-Load4 can effectively predict general psychopathology scores. The general psychopathology scale primarily reflects the patient's cognitive, and behavioral dysfunction, which greatly impacts the patient's daily life and cognitive functioning. The above results suggest that SAN1-Load4 can effectively predict the patient's pathological symptoms and cognitive functions. Although different experimental tasks were employed, Lad et al. (2024) also found that non-verbal auditory memory can predict general cognitive abilities.

Of note, we didn't find an obvious group difference in SAN1. Two explanations could be considered. First, as the early stage of memory maintenance, the patients` ability to maintain memory items is relatively intact, thus showing less group difference. This is consistent with the result that the early stage of maintenance shows a significantly larger SAN load effect compared to the later stage of maintenance (Lefebvre and Jolicœur, 2016). Similar findings have also been found in our results. Second, there is the potential impact of individual differences. Lefebvre and Jolicœur (2016) found that even healthy individuals show a wide range of individual difference in memory capacity. Some subjects have a maximum memory span of only one item, while others can remember up to four items. This individual difference in memory span is further reflected in the amplitude of the SAN1 and ultimately becomes a potential interfering factor (Lefebvre and Jolicœur, 2016). Replication of these findings in an independent data sample in future studies is needed.

## 5 Limitations

Several limitations must be acknowledged. Firstly, since the behavioral data includes only one female subject and the EEG data lacks any female subjects, the current results primarily reflect the performance of male subjects. Secondly, since almost all of the SZ received medications, the treatment effect cannot be ruled out. However, whether antipsychotics have an effect on auditory ERP is still controversial (e.g., Ford et al., 1994; Umbricht et al., 1998), and future research can solve this problem through large-scale longitudinal follow-up studies. Finally, it is necessary to reveal the dynamic changes in non-verbal auditory WM of SZ through time-frequency analysis and brain network analysis.

## 6 Conclusion

In summary, current research indicates that SZ exhibit significant impairments in non-verbal auditory WM. Compared to HC, patients not only have lower accuracy but also demonstrate slower reaction times. During the retention phase, their memory maintenance decays rapidly, exhibiting a markedly smaller frontal SAN2. Moreover, the SAN1 is significantly correlated with their general pathological symptoms. SAN1-Load 4 can effectively predict the pathological symptoms and cognitive dysfunctions.

## CRediT authorship contribution statement

**Lei Liu:** Writing – review & editing, Writing – original draft, Investigation, Formal analysis, Conceptualization. **Wenyang Han:** Writing – review & editing, Investigation. **Juntao Yu:** Writing – review & editing, Investigation. **Lingna Lou:** Writing – review & editing, Writing – original draft. **Dewen Zhou:** Writing – review & editing, Writing – original draft. **Liang Li:** Funding acquisition, Conceptualization. **Peng Xu:** Writing – review & editing, Writing – original draft, Funding acquisition. **Feng Zou:** Writing – review & editing, Writing – original draft, Conceptualization.

## Declaration of competing interest

The authors have no conflict of interest to declare.

## Data Availability

The raw data supporting the conclusions of this article will be made available by the authors on request. (windzou1974@163.com) upon request.
